# Integrated primary care, the collaboration imperative inter-organizational cooperation in the integrated primary care field: a theoretical framework

**Published:** 2012-09-04

**Authors:** Pim P Valentijn, Marc A Bruijnzeels, Rob J de Leeuw, Guus J.P Schrijvers

**Affiliations:** Researcher, Jan van Es Institute, Netherlands Expert Centre Integrated Primary Care, The Netherlands; Director, Jan van Es Institute, Netherlands Expert Centre Integrated Primary Care, The Netherlands; Program Leader Mental Health, Julius Center for Health Sciences and Primary Care, Division Public Health, Utrecht University, The Netherlands; Professor Structure and Functioning of the Health Care Division, Julius Center for Health Sciences and Primary Care, Utrecht University, The Netherlands

**Keywords:** primary care, integrated care, inter-organizational relations, collaboration, concepts, terminology, complexity

## Abstract

**Purpose:**

Capacity problems and political pressures have led to a rapid change in the organization of primary care from mono disciplinary small business to complex inter-organizational relationships. It is assumed that inter-organizational collaboration is the driving force to achieve integrated (primary) care. Despite the importance of collaboration and integration of services in primary care, there is no unambiguous definition for both concepts. The purpose of this study is to examine and link the conceptualisation and validation of the terms inter-organizational collaboration and integrated primary care using a theoretical framework.

**Theory:**

The theoretical framework is based on the complex collaboration process of negotiation among multiple stakeholder groups in primary care.

**Methods:**

A literature review of health sciences and business databases, and targeted grey literature sources. Based on the literature review we operationalized the constructs of inter-organizational collaboration and integrated primary care in a theoretical framework. The framework is being validated in an explorative study of 80 primary care projects in the Netherlands.

**Results and conclusions:**

Integrated primary care is considered as a multidimensional construct based on a continuum of integration, extending from segregation to integration. The synthesis of the current theories and concepts of inter-organizational collaboration is insufficient to deal with the complexity of collaborative issues in primary care. One coherent and integrated theoretical framework was found that could make the complex collaboration process in primary care transparent. This study presented theoretical framework is a first step to understand the patterns of successful collaboration and integration in primary care services. These patterns can give insights in the organization forms needed to create a good working integrated (primary) care system that fits the local needs of a population. Preliminary data of the patterns of collaboration and integration will be presented.

Powerpoint presentation available at http://www.integratedcare.org at congresses – San Marino – programme.

